# Cohort Profile: Genetics of Diabetes Audit and Research in Tayside Scotland (GoDARTS)

**DOI:** 10.1093/ije/dyx140

**Published:** 2017-09-07

**Authors:** Harry L Hébert, Bridget Shepherd, Keith Milburn, Abirami Veluchamy, Weihua Meng, Fiona Carr, Louise A Donnelly, Roger Tavendale, Graham Leese, Helen M Colhoun, Ellie Dow, Andrew D Morris, Alexander S Doney, Chim C Lang, Ewan R Pearson, Blair H Smith, Colin N A Palmer

**Affiliations:** 1 Division of Population Health Sciences; 2 Pat Macpherson Centre for Pharmacogenetics and Pharmacogenomics; 3 Health Informatics Centre Services, Ninewells Hospital & Medical School, University of Dundee, Dundee, UK; 4 Institute of Genetics & Molecular Medicine; 5 Usher Institute of Population Health Sciences and Informatics, University of Edinburgh, Edinburgh, UK

## Why was the cohort set up?

The prevalence of diabetes worldwide has been steadily increasing over the past 20 years. In 1997 it was estimated to be 124 million,^1^ in 2015 it was estimated to be 415 million among 20–70 year olds, and this is expected to rise to 642 million by 2040.^2^ In the UK, an estimated 4 million people have diabetes either diagnosed or undiagnosed.^2^ This represents a significant burden on health care resources,^3^ particularly given that type 2 diabetes (T2D) is associated with comorbidities including obesity,^4^ cardiovascular disease,^5^ chronic kidney disease^6^ and neuropathy.^7^ T2D is a complex disorder, caused by a combination of environmental and genetic factors.^8^ Before the first genome-wide association study (GWAS) was conducted for T2D in 2007,^9^ very few genetic loci were known to be involved with T2D. However, linkage and candidate-gene association studies have often failed to replicate findings through lack of power and inadequate knowledge of the underlying biological pathways.^10^^,^^11^

Diabetes Audit and Research in Tayside Scotland (DARTS) started in 1996 as a joint collaboration between the University of Dundee’s Department of Medicine and Medicines Monitoring Unit (MEMO), three Tayside Health Care Trusts (at Ninewells Hospital and Medical School, Perth Royal Infirmary and Stracathro Hospital) and a group of Tayside general practitioners (GPs) with a special interest in diabetes care.^12^ Initially supported by the Scottish Home and Health Department, the Wellcome Trust, the Robertson Trust and Tenovus Tayside, the aim of the study was to identify all patients with diabetes within the wider Tayside region, through electronic record linkage, in order to improve health care over and above that which was practical through existing general practice lists alone. In 1998, consenting patients within this electronic database were recruited to the Genetics of DARTS (GoDARTS) study and invited to provide a blood sample for DNA extraction, for research purposes. At the same time, they were invited to provide phenotypic data (clinical and lifestyle factors), through questionnaires and clinical examination. This resource was intended to help identify the relative contribution of specific genetic and environmental factors that are associated with disease onset, progression and response to treatment.^10^^,^^11^

## Who is in the cohort?

Patients from the Tayside region of Scotland (*n* = 391 274 on 1 January 1996^12^) were added to the DARTS register, for clinical purposes, through electronic record linkage on the basis of having diabetes mellitus according to primary and/or secondary care data sources. These included hospital diabetes clinics, mobile diabetes eye units, diabetes prescription databases, the Tayside regional biochemistry database and all diabetes-related hospital discharge records. This electronic record linkage technique has a sensitivity of 97% and is continually being updated, creating a longitudinal dataset of clinical data which is manually validated by a dedicated team of clinicians.^12^ Patients with T2D, which comprises around 90% of all diabetes cases, were invited to participate in the GoDARTS study either at diabetes or eye screening clinics or through their GP.

For the pilot phase of the study (GoDARTS1), 2763 patients with T2D were recruited from December 1998 to October 2004. This phase was used to test recruitment processes and the ability to anonymously link patient clinical data from electronic records to the study, and was funded by Tenovus Tayside. As this was the primary aim of the pilot phase, only blood samples were taken at the point of recruitment and no baseline data were recorded.

**Figure 1:**
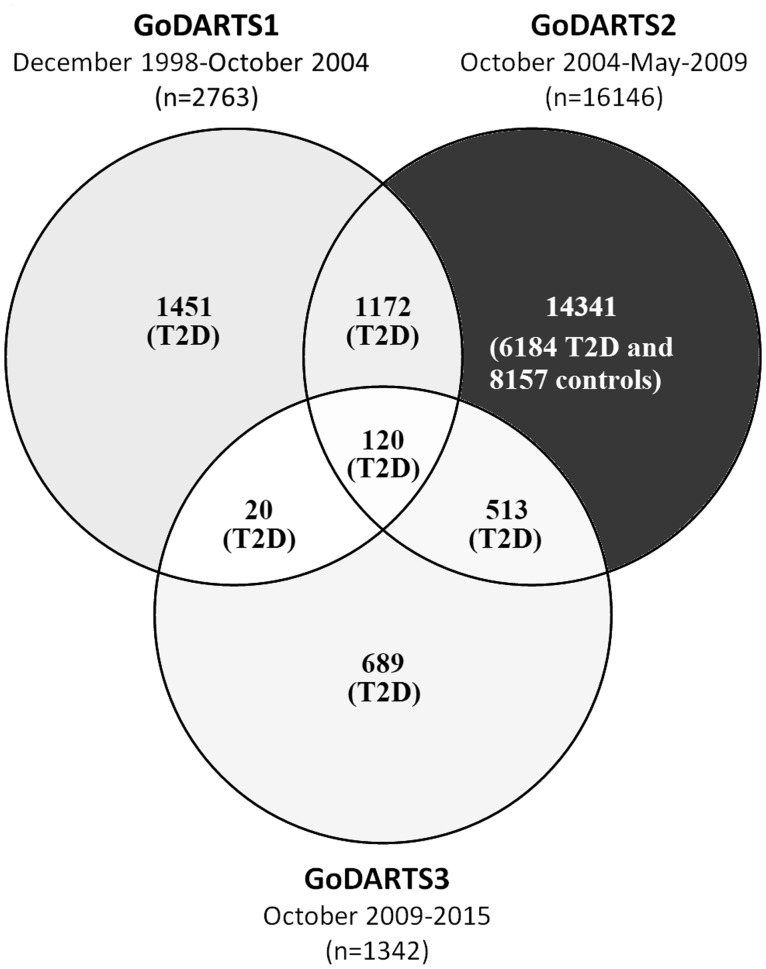
A venn diagram showing the overlap in patient recruitment between GoDARTS1, GoDARTS2 and GoDARTS3.

From October 2004 to May 2009, a second collection (GoDARTS2) was undertaken as part of the Wellcome Trust United Kingdom Type 2 Diabetes Case-Control Collection (WTCCC). A total of 16 146 people were recruited in this phase, including 7989 patients with T2D and 8157 matched healthy controls. We initially invited five matched non-diabetic controls per case from the corresponding GP practice; however, after initial success, this was reduced to two controls per case and on average one of the invited controls accepted. This incidentally included 1292 patients with T2D who had already been recruited in the GoDARTS1 phase. Baseline clinical and lifestyle measurements (Table 1) were recorded for all patients recruited in GoDARTS2. From October 2009 until 2015, an extension to the WTCCC project was granted (GoDARTS3), with 1342 patients with T2D being recruited during this time. Some of these participants had also been recruited to GoDARTS1 (*n* = 20), GoDARTS2 (*n* = 513) or both (*n* = 120), where baseline data did not exist or original DNA quality was poor (Figure 1). This gives a current total GoDARTS cohort of 18 306 participants, 10 149 of whom have T2D ( ∼ 44.8% of the DARTS study, representing the diabetic population in Tayside) and 8157 of whom were healthy controls at baseline.

**Table 1:** Summary of baseline data collected and comparison of response rates between cases and controls in GoDARTS

**Measure**	**Response** (%)		**Notes**
	**Cases** (*n* = 8698)	**Controls** (*n* = 8140)	
Age (years)	99.37	99.53	
Gender	100	100	
Ethnicity	100	100	Caucasian or non-Caucasian
Height (cm)	99.68	100	
Weight (kg)	99.57	99.95	
Waist (cm)	99.57	99.84	
Diastolic blood pressure (mmHg)	1 = 99.90	1 = 99.99	Two measures taken
	2 = 99.17	2 = 99.83	
Systolic blood pressure (mmHg)	1 = 99.90	1 = 99.99	Two measures taken
	2 = 99.17	2 = 99.83	
Heart rate (bpm)	1 = 99.75 2 = 98.87	1 = 99.90 2 = 99.50	Two measures taken
Diabetes treatment	99.21	n/a	Diet, tablets or pills
Diabetes medication^a^	4.73	n/a	Dose, date and time last taken
Family history of diabetes^a^	8.24	n/a	
Present smoking status (Amount smoked)	99.71 (85.77)^b^	99.94 (81.80)^b^	
Past smoking status (Amount smoked)	99.61 (95.05)^b^	99.90 (95.22)^b^	
Age started smoking	99.22^c^	99.52^c^	
Stopped normal periods (Age periods stopped)	99.53 (90.70)^b^	99.85 (73.85)^b^	
Level of physical activity during work:			
1. Recently	1 = 99.66	1 = 99.89	
2. Past 10 years	2 = 99.69	2 = 99.85	
3. Youth	3 = 99.56	3 = 99.93	
Level of physical activity during travel:			
1. Recently	1 = 99.60	1 = 99.89	
2. Past 10 years	2 = 99.59	2 = 99.84	
3. Youth	3 = 99.47	3 = 99.84	
Level of physical activity during leisure:			
1. Recently	1 = 99.67	1 = 99.93	
2. Past 10 years	2 = 99.57	2 = 99.82	
3. Youth	3 = 99.54	3 = 99.91	
Confirmed type 2 diabetes	99.25	n/a	
Location patient was screened	100	100	Clinic, GP surgery, home or other
HbA1c (%)	93.17	99.82	
Cholesterol (mmol/l)	92.73	99.94	
HDL (mmol/l)	92.72	99.94	
LDL (mmol/l)	84.07	97.02	
Creatinine (µmol/l)	92.99	99.84	
Triglycerides (mmol/l)	92.62	99.84	

n/a, not available.

^a^Baseline data only available in participants recruited in GoDARTS3.

^b^Response rate calculated according to the number of positive responses to the main question.

^c^Response rate calculated according to the number of positive responses to present and/or past smoking status.

Currently the cohort is in the early stages of GoDARTS4, the fourth phase of the study. In this phase, recruitment is continuing through a number of initiatives including the Scottish Health Research Register (SHARE)/Scottish Diabetes Research Network (SDRN), the Genetics of SHARE (GoSHARE) and GoDARTS-Scotland. SHARE/SDRN is a register of patients in Scotland who want to participate in medical research and have provided consent for their electronic medical records to be used for research purposes [http://www.share-sdrn.org]. GoSHARE is a parallel project which additionally aims to get permission to collect spare blood from people attending for routine clinical tests at hospital or GP clinics, that would otherwise to go to waste after the necessary tests had been performed [http://www.goshare.org.uk/]. Since the aim is to involve everyone who is resident in the Tayside area, this will inevitably include people with T2D, and they will contribute to the GoDARTS study. GoDARTS Scotland is a sub-study specifically recruiting people who have been diagnosed with T2D in the past 2 years in order to study response to therapies, including metformin.

At the point of recruitment, all participants in GoDARTS provide, by invitation, informed consent for their data to be used for research purposes and explicit consent for use in collaboration with industry. This includes allowing their baseline data to be linked anonymously to individual patient medical records including laboratory data, hospital admissions and Scottish Care Information – Diabetes (SCI-Diabetes) data. SCI-Diabetes is a shared electronic patient record which can be accessed by health professionals and researchers to aid the treatment of diabetes patients in Scotland. In this way, longitudinal data can be accessed relating to routine diabetes management, for example glycosylated haemoglobin (HbA1c), fasting insulin and fasting glucose, as well as previous patient diagnoses including diabetic complications. Furthermore, ∼ 95% of patients have consented to being contacted for future studies, aiding research beyond T2D.

## How often have they been followed up?

As patients attend a baseline clinic at recruitment, initial measurements are cross-sectional. However, the use of electronic record linkage, which automatically updates patient details and grants access to NHS data as far back as 1987, makes GoDARTS a longitudinal cohort. This is made possible through the use of the community health index (CHI) number, which is a unique numerical identifier issued to each patient on first registration with a GP or admission to a hospital in Scotland. Around 96% of the UK population are estimated to be registered with a GP.^13^ The CHI is a 10-digit number consisting of six digits corresponding to the patient’s date of birth (DDMMYY), two digits randomly generated, one digit corresponding to the patients gender (odd for males, even for females) and one check digit. The CHI number links to live databases which are constantly being updated, such as the Scottish Morbidity Record (SMR) providing data on primary and secondary diagnoses for patients discharged from hospital since 1980, the Tayside echocardiography database providing data on all echocardiograms performed at Ninewells Hospital since 1994, the General Registrar’s Office providing mortality data since 1998, the biochemistry database listing all assays performed since 1981 and a database containing all prescriptions dispensed since 1989. This allows identification of an up-to-date record of every individual’s health care processes and outcomes and linkage of corresponding datasets. An anonymization process converts the CHI into a study pro-CHI, to protect the identities and confidentiality of individuals while retaining the ability to link patient data across multiple datasets.

## What has been measured?

For GoDARTS1, only blood samples were taken for DNA extraction as this was a pilot phase used to test the ability to link electronic health records anonymously to genetic data. For GoDARTS2 and GoDARTS3, participants completed a lifestyle questionnaire and consented to baseline measurements being recorded at recruitment (Table 1). In addition, during GoDARTS3 urine samples ( ∼ 80% of recruits) were taken for proteomic and metabolomics analysis and RNA ( ∼ 30% of recruits) was extracted from blood samples. The lifestyle questionnaire contains items relating to smoking history (present and past status, along with amount and age started where applicable), as well as level of physical activities in three common locations (work/education, travel and home life) over three different time periods in life (recently, past 10 years and youth). In addition, women were asked about their menopausal history. Baseline observations were recorded and included height, weight and waist measurements, as well as heart rate and blood pressure. The patient’s recruitment information was recorded including ethnicity, screening location, confirmation of T2D and medication history, as well as family history of diabetes and whether the patient had previously participated in GoDARTS. As baseline data were only recorded for participants recruited in GoDARTS2 and GoDARTS3, there are 1451 participants who were only involved in GoDARTS1 (Figure 1) and do not have these data. Furthermore, there are 17 healthy control participants from GoDARTS2 who are missing baseline data, meaning that a total of 16 838 patients have these available, including 8698 cases and 8140 controls.

**Figure 2:**
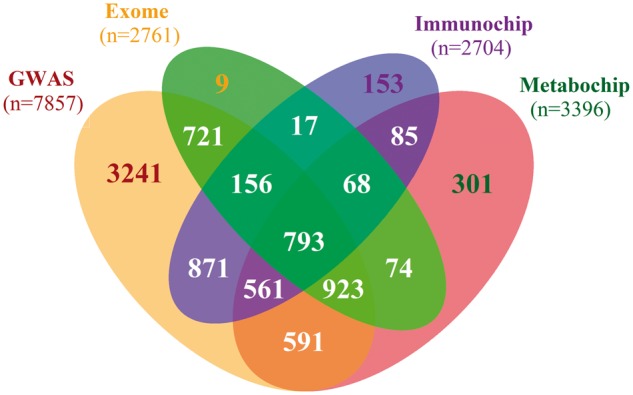
A venn diagram showing the overlap of T2D cases genotyped between different platforms. Overall, 8564 cases out of a possible 10 149 have been genotyped on at least one platform, with 7857 having genome-wide data.

**Figure 3:**
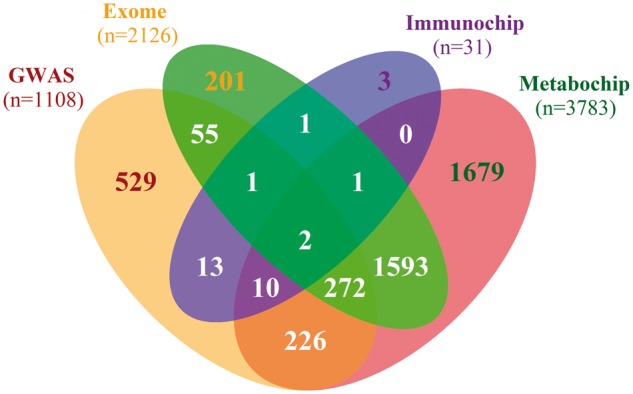
A venn diagram showing the overlap of controls genotyped between different platforms. Overall, 4586 controls out of a possible 8157 have been genotyped on at least one platform, with 1108 having genome-wide data.

As well as phenotypic data, genetic data are available for 8564 T2D cases (Figure 2) and 4586 controls (Figure 3) after quality control. Samples have been genotyped across five platforms. GWAS data have been obtained for 7857 T2D cases and 1108 controls, using the Affymetrix Genome-Wide Human SNP Array 6.0 and the Illumina HumanOmniExpress. The Affymetrix GWAS chip contains 932 979 single nucleotide polymorphisms (SNPs), and the Illumina GWAS chip contains 731 296. This has allowed for imputation of additional and missing genotypes by SHAPEIT^14^ and IMPUTE2^15^ using the 1000 Genomes reference panel.^16^ In addition, 707 T2D cases and 3478 controls have been genotyped using custom genotyping arrays from Illumina. These include the Immunochip, Cardio-Metabochip (Metabochip) and Human Exome array. The Immunochip contains 196 524 genetic markers from loci that have previously been associated with at least one of 13 autoimmune diseases, including T1D,^17^ and the Metabochip contains 196 725 SNPs from loci that have prior evidence of association with T2D, coronary artery disease/myocardial infarction and 21 related traits.^18^ The specific criteria by which markers on the Cardio-Metabochip and the Immunochip have been chosen makes these platforms a cost-effective means of replicating and fine-mapping known loci and discovering novel loci by virtue of overlapping biological mechanisms between the related traits. The Human Exome Array contains 247 870 genetic markers from across the exome, allowing for studies to focus on identifying protein-altering variants.^19^

## What has it found?

### Baseline and follow-up epidemiology

Baseline clinical and demographic statistics are summarized in Table 2. Overall, 53.33% of the cohort are male, which is similar to the proportion represented in DARTS (52.83%), with the proportion being higher in cases (56.38%) compared with controls (50.08%). The majority of the cohort are Caucasian (99.70%) and the median age at recruitment was higher in cases (67 years) compared with controls (60 years). This observation is also apparent when the cases and controls are further dichotomized into males (66 vs 62 years) and females (68 vs 58 years). The cohort contains data on a number of continuous traits known to be associated with T2D. For example, median body mass index (BMI) (30.6 vs 26.6 kg/m^2^), resting heart rate (1st = 73 vs 68 bpm), creatinine (89 vs 87 µmol/l) and triglyceride (1.880 vs 1.315 mmol/l) levels were all higher in cases compared with controls. Furthermore, there was a higher proportion of past smokers among those with T2D (63.14% vs 53.56%).

**Table 2:** Comparison between cases and controls in baseline measurements

**Measure**	**Cases***n* = 8698	**Controls***n* = 8140	**Overall**
Gender (% male)	56.38	50.08	53.33
Age (years)	67	60	64
Males	66	62	65
Females	68	58	63
Ethnicity (% Caucasian)	99.68	99.72	99.70
BMI (median) (kg/m^2^)	30.6	26.6	28.4
Males	30.0	27.0	28.4
Females	31.5	26.2	28.4
Height (cm)	168	168.5	168
Males	174	175	175
Females	159	162	160
Weight (kg)	86.05	76.20	81.3
Males	90.4	83.2	87
Females	79.8	68.5	73
Waist (cm)	104	93	99
Males	106	98	102
Females	101	86	93
Current smokers (%)	16.37	16.35	16.36
Males	16.42	16.49	16.45
Females	16.31	16.20	16.25
Current amount smoked (packs per week)	5	5	5
Males	6	5	5
Females	5	5	5
Past smokers (%)	63.14	53.56	58.50
Males	70.24	59.80	65.49
Females	53.97	47.30	50.52
Past amount smoked (packs per week)	7	7	7
Males	7	7	7
Females	7	6	7
Age started smoking (years)	16	16	16
Males	16	16	16
Females	17	17	17
Resting pulse 1 (bpm)	73	68	70
Males	72	66	69
Females	75	70	72
Resting pulse 2 (bpm)	73	68	71
Males	72	66	69
Females	75	70	72
Systolic blood pressure 1 (mm Hg)	141	135	139
Males	141	138	140
Females	141	131	136
Diastolic blood pressure 1 (mm Hg)	77	80	78
Males	78	81	79
Females	76	78	77
Systolic blood pressure 2 (mm Hg)	140	134	137
Males	140	137	139
Females	140	130	135
Diastolic blood pressure 2 (mm Hg)	75	78	77
Males	76	80	78
Females	74	77	76
HbA1c (%)	7.1	5.5	6.0
Males	7.1	5.5	6.0
Females	7.2	5.5	5.9
Cholesterol (mmol/l)	4.30	5.24	4.72
Males	4.19	5.12	4.57
Females	4.44	5.36	4.90
HDL (mmol/l)	1.28	1.57	1.41
Males	1.21	1.41	1.30
Females	1.39	1.76	1.57
LDL (mmol/l)	2.01	2.93	2.43
Males	1.97	2.90	2.37
Females	2.05	2.97	2.51
Creatinine (µmol/l)	89	87	88
Males	95	95	95
Females	82	79	80
Triglycerides (mmol/l)	1.880	1.315	1.59
Males	1.87	1.47	1.69
Females	1.88	1.19	1.49

Median values given for all continuous data.

**Figure 4:**
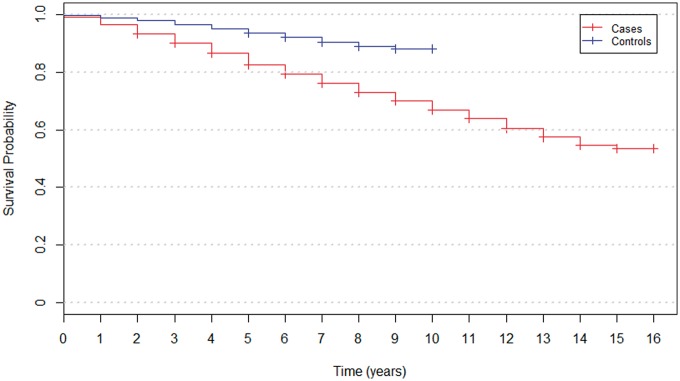
A Kaplan-Meier plot comparing survival rate since baseline recruitment in cases and controls.

As of 2014, mortality data have shown that the number of deaths at 9 years after recruitment was 2587 out of 10 149among the cases and the Kaplan–Meier survival probability is 70.0%, whereas among the controls the number of deaths was 851 out of 8157 and the Kaplan–Meier survival probability is 88.2% (Figure 4). Control group mortality data do not go beyond this, as recruitment of controls did not begin until GoDARTS2 (approximately 7 years after the start of GoDARTS1); however, the number of deaths after 16 years among the cases was 2941 (out of 10 149) with a Kaplan–Meier survival probability of 53.5%.

**Figure 5:**
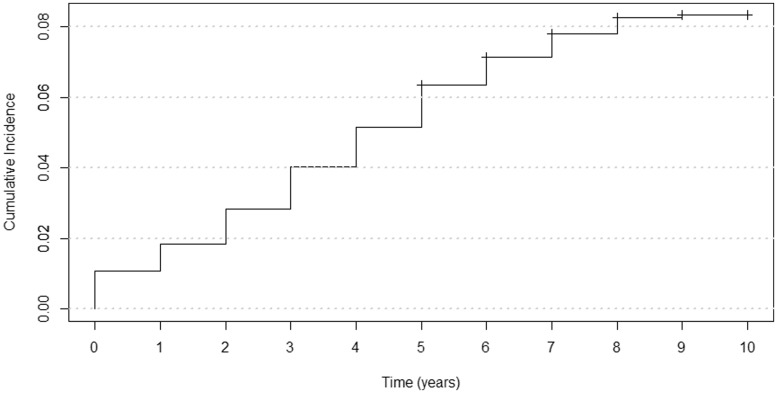
A Kaplan-Meier cumulative incidence plot of diabetes in the GoDARTS baseline controls group.

**Figure 6:**
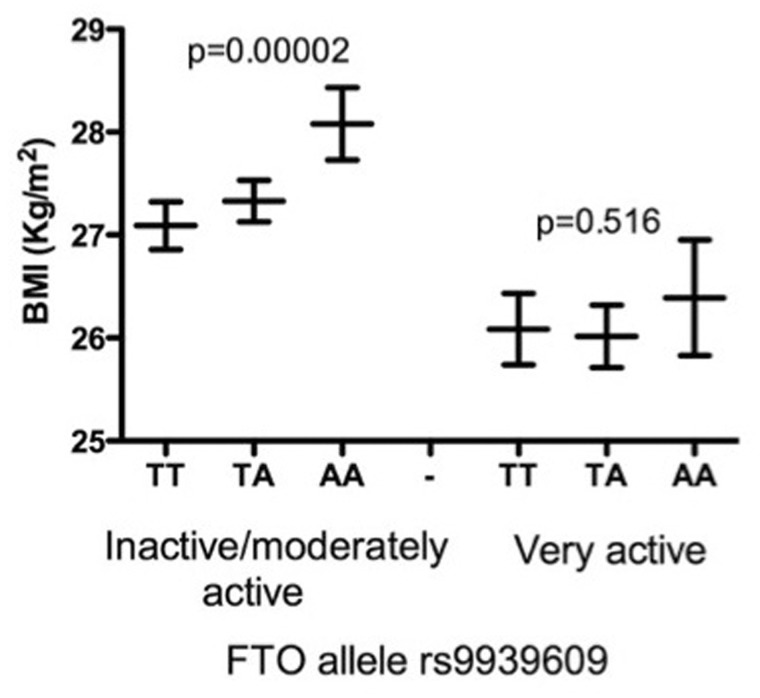
A plot showing that increased physical activity successfully stratifies the association of the obesity risk allele rs9939609 in *FTO* with BMI.

According to SCI-Diabetes data, the number of people initially recruited as controls at baseline, but who went on to develop diabetes, is 650 (out of 8157) and the Kaplan–Meier cumulative incidence probability is 8.3% (Figure 5). Also captured were self-reported physical activity data, and these can be seen to successfully stratify the effect of the fat mass and obesity-associated protein gene (*FTO*) risk allele, rs9939609, where the genetic association with BMI is largely attenuated in active individuals, as has been observed in large meta-analyses (Figure 6).

### Research output

Over 100 studies have been published using GoDARTS either as the primary study cohort or as part of a larger meta-analysis or replication. The following is a summary of important studies, in all of which GoDARTS has been involved. A full and up-to-date list of studies can be found at [www.researcherid.com/rid/K-9448‐2016] (ResearcherID: K-9448‐2016).

GoDARTS began in the pre-GWAS era, with its first study being published in 2002.^20^ At this time candidate-gene studies were conducted, and these were particularly successful in replicating associations at the peroxisome proliferator-activated receptor (PPAR) transcription factor family, which had previously been associated with a range of phenotypes including T2D. In particular, two variants (rs1801282 and rs3856806) in *PPARG* were shown to have opposing effects on body weight, with the non-synonymous rs1801282 (Pro12Ala) associated with lower BMI and the synonymous rs3856806 (C1431T) associated with higher BMI.^20^ This provided an explanation for previous inconsistencies found between this gene and BMI. Similar findings were reported with respect to T2D susceptibility^21^ and myocardial infarction,^22^ with haplotype analysis confirming the protective effect of rs1801282 with these phenotypes in contrast to the risk effect of rs3856806. In addition, rs1801282 was shown to have an opposing effect to another polymorphism, rs10865710 (C681G), with respect to height and weight in pre-pubertal children.^23^ Further studies identified similar attenuations in the related genes *PPARA* with myocardial infarction,^24^ and replicated an association at *PPARD* with reduced adult height.^25^

In 2007, the GoDARTS study became part of the UK T2D genetics consortium collection (UKT2DGCC), which formed the main replication cohort for the WTCCC. This involved large-scale studies investigating quantitative traits including height and obesity, in addition to T2D. One of the first published studies using the UKT2DGCC found an association in the *FTO* gene with obesity. This effect was observed from age 7 years and older.^26^ Another related study identified associations at the *MC4R* gene with BMI and childhood obesity among 7–11-year-olds.^27^ GoDARTS was involved in the seminal publications describing the identification of many genes for type 2 diabetes, including the first descriptions of *CDKAL1* and *CDKN2A/CDKN2B* variants and risk for diabetes^28–32^ and also the original report of the association of the *HMGA2* gene with height.^33^^,^^34^ Smaller-scale candidate-gene studies have also been conducted to good effect in T2D, as demonstrated by the identification of the *WFS1* gene.^35^ T2D-related traits have also been studied with 10 novel loci associated with fasting glucose levels,^29^^,^^36^ IGF1 influencing fasting insulin^29^ and GIPR associated with 2-h glucose levels after oral glucose challenge.^32^

In addition to GWAS of anthropometric traits and T2D susceptibility, GoDARTS has pioneered the use of electronic medical records for the study of drug efficacy. This was initially used to study response to sulphonylureas^37^ and statin lipid-lowering agents.^38^ As part of the WTCCC2 consortium, GoDARTS served as a discovery cohort for the GWAS of response to statins and metformin.^39^ The metformin analysis found a novel association of glycaemic response to metformin at a locus including the ataxia telangiectasia mutated (*ATM*) gene, which provided new clues to the mechanism of action of this mysterious drug.^40^^,^^41^ More recently, the GoDARTS cohort was the main discovery cohort for a large Metformin Genetics Consortium study (MetGen) that showed a variant in *SLC2A2* to be consistently associated with altered glycaemic response to metformin.^42^ Other investigated phenotypes in GoDARTS include response to thiazolidinediones^43^ and sulphonylureas,^37^^,^^44^ and adverse reactions to statins, metformin and angiotensin-converting enzyme (ACE) inhibitors.^40^^,^^45^

Linkage of the GoDARTS study to the Tayside echocardiography database has allowed the identification of genetic variants associated with left ventricular hypertrophy,^46^ as well as the association of both high ( > 10%) and low ( < 6%) HbA1c levels with risk of incident heart failure.^47^

From around 2012, the focus of studies has switched from genome-wide analysis to more targeted approaches. One of these methods involves genotyping with the Metabochip. This has been influential in the discovery and fine-mapping of loci in diabetes- and cardiovascular disease-related phenotypes. In particular, 17 novel T2D loci^48^^,^^49^ have been discovered and a further 39 genetic regions have undergone variant localization and genomic annotation to identify the causative mutations.^50^ Similar success has been seen in coronary artery disease, with 25 loci being identified.^51^^,^^52^ Glycaemic and anthropometric traits have also been studied successfully, with loci being identified with fasting insulin, fasting glucose, height and obesity.^53–57^

Another targeted approach that has been increasingly used is exome sequencing and follow-up studies using the Illumina Exome chip. This has been used to capture rare disease-associated variants lying within the protein coding region of the genome, which are hypothesized to make up much of the missing heritability in common diseases. Due to their protein-altering nature, disease-associated polymorphisms discovered in these regions are likely to be causative, and the biological pathways can be more easily elucidated than other study methods. Exome chip genotyping in GoDARTS contributed to the demonstration that, loss-of-function variants in *APOC3*^58^ and *ANGPTL4*^59^ are simultaneously associated with lower triglyceride levels and reduced risk of coronary artery disease (CAD). Similarly, inactivating mutations in *NPC1L1* have a protective effect on CAD risk and reduced low-density lipoprotein (LDL) cholesterol level.^60^ Whereas other phenotypes related to glycaemic traits,^61^^,^^62^ lipids^63^ and adiponectin^64^ have successfully identified loci, a large exome sequencing/exome chip study has revealed that coding variation does not play a large role in T2D susceptibility.^65^

In more recent years, GoDARTS has been used in Mendelian randomzsation studies to establish the causal relationship between a variable and disease. This has been used to demonstrate a role for sex hormone-binding globulin^66^ and to rule out a causal effect between circulating triglycerides and adiponectin in T2D.^67^^,^^68^ In addition, adiposity and adiponectin have been implicated in moderating CVD risk.^69^^,^^70^ Other studies have been conducted, and a more comprehensive list of disease phenotypes can be found in Table 3.

**Table 3:** Summary of phenotypes studied using the GoDARTS cohort

Phenotype	Study reference
ACEi-induced cough	(71)
Adiponectin levels	(64)
ARMD and diabetic retinal disease in T2D	(72)
Diabetic atherogenic lipid profile and myocardial infarction	(73)
Diabetic chronic kidney disease	(74)
Diabetic glomerular filtration rate	(75)
Diabetic left ventricular hypertrophy	(46)
Diabetic myocardial infarction	(76, 77)
Diabetic nephropathy	(78)
Diabetic neuropathic pain	(79, 80)
Diabetic retinopathy	(81)
Diabetic smoking-related cardiovascular morbidity	(82)
Diabetic statin intolerance	(83)
Glycaemic response to metformin	(84–86)
Glycaemic response to sulphonylureas	(37, 87, 88)
Glycaemic traits	(29, 61, 89)
Intolerance to metformin in T2D	(90)
LDLc response to statin therapy and CAD during statin treatment	(91)
Lipid response to statin therapy	(40, 45, 92)
Prostate cancer	(93)
Serum triglyceride level, insulin resistance and T2D in severe obesity	(94)
Serum urate concentration, excretion and gout	(95)
Stroke in T2D	(96)
T2D	(31, 35, 50, 98)

ACEi, angiotensin-converting enzyme inhibitor; ARMD, age-related macular degeneration; CAD, coronary artery disease; LDLc, low-density lipoprotein cholesterol; T2D, type 2 diabetes.

## What are the strengths and weaknesses?

The main strengths of GoDARTS are: its large size [including 10 149 participants with T2D ( ∼ 51% of people with T2D in Tayside) and 8157 controls]; the availability of rich genetic and phenotypic data; the ability to link patient genetic and baseline data to routine electronic medical records; and the existing consent for use of these for research and for future contact for possible research participation, especially the potential for recruitment by genotype studies. Consent for future contact has allowed further studies to take place. One of these is DOLORisk, an EU Horizon 2020-funded project that will be re-phenotyping participants for neuropathic pain and related traits, to identify possible risk factors [http://dolorisk.eu/]. As a result, the GoDARTS cohort is rich in longitudinal phenotypic data, such as biochemistry, prescribing, morbidity and demography. In addition the linkage of the study to participants’ individual electronic medical records, which are constantly updated, means the cohort is not limited by loss-to-follow up bias that can beset other longitudinal studies. This linkage is made possible through the use of the CHI number which allows patient data to remain anonymous. The availability of a large range of clinical and demographic data allows a large range of diabetes-related phenotypes to be investigated, on both genome-wide and more targeted scales, and also provides a means to control for common confounders such as BMI, smoking, age and blood pressure. The large number of samples recruited at baseline (8697 T2D cases and 8141 controls) provides the statistical power with which to identify genetic variants conferring susceptibility to disease, both as a stand-alone cohort and as part of meta-analyses. Furthermore, RNA has been collected from patient blood samples, which will enable gene expression studies to be conducted in the future.

A weakness of the GoDARTS cohort is the missing baseline data of GoDARTS1 patients who were not subsequently recruited again in GoDARTS1 or 2. This is due to GoDARTS1 being a pilot phase of the study, and consequently baseline data were not collected at this stage. However, linkage is still possible for these samples. Patients were also not necessarily recruited at the point of diagnosis, which creates heterogeneity in the effects of disease duration on the serum samples obtained. Another weakness concerns the lifestyle questionnaire that was administered to patients. As this was self-completed by the patients, physical activity, smoking and female menopausal history are subject to recall bias.

## Where can I find out more?

Further information about this cohort can be found at [http://diabetesgenetics.dundee.ac.uk/]. Access to the dataset is available to researchers worldwide and access requests, together with the general management of the resource, are handled by the Access Group. More details on the application and collaboration process can be found at [http://diabetesgenetics.dundee.ac.uk/Community.aspx].

Profile in a nutshell

- GoDARTS was set up in 1998 in order study the genetics underpinning type 2 diabetes (T2D) susceptibility, diabetes complications and patient response to therapy.
- The study is a branch of the pre-existing Diabetes Audit and Research in Tayside Scotland study, which was set up to identify all patients within the Tayside area with diabetes, through electronic record linkage, to provide better care over and above existing registries.
- As of 2014, the study has 18 306 participants aged 16–98, of whom 10 149 have T2D and 8157 are controls. Baseline data are available for 16 838 (8698 cases and 8140 controls), and 8564 T2D cases and 4586 controls have genetic data.
- Baseline data collection includes a self-completed lifestyle questionnaire containing items on physical activity, smoking history, and menopausal history for women. In addition, clinical observations were recorded and blood and urine samples were taken. Baseline data are linked to existing NHS records, providing morbidity, mortality and prescribing data by electronic record linkage to enable long-term follow-up.
- Consent has been provided by ∼ 95% of participants to be re-contacted regarding possible participation in future studies. Information on collaboration and data access can be found at [http://diabetesgenetics.dundee.ac.uk/]. A list of GoDARTS publications can be found at [www.researcherid.com/rid/K-9448‐2016].

## Funding

The Wellcome Trust United Kingdom Type 2 Diabetes Case Control Collection (supporting GoDARTS) was funded by the Wellcome Trust (072960/Z/03/Z, 084726/Z/08/Z, 084727/Z/08/Z, 085475/Z/08/Z, 085475/B/08/Z) and as part of the EU IMI-SUMMIT programme. C.P. has received grant funding from the Wellcome Trust to develop the GoDARTS cohort. E.P. holds a WT New Investigator award (102820/Z/13/Z). B.H.S., C.N.A.P. and W.M. are members of the DOLORisk consortium which is funded by the European Commission Horizon 2020 (ID:633491), and are partly supported by this grant. H.L.H. and A.V. are supported by DOLORisk.
